# Supplementary figures and images for: Head-to-Head Comparison of Two Nomograms Predicting Probability of Lymph Node Invasion in Prostate Cancer and the Therapeutic Impact of Higher Nomogram Threshold
Source: J Clin Med. 2021 Mar 2;10(5):999. doi: 10.3390/jcm10050999 (PMC7957888; doi:10.3390/jcm10050999)

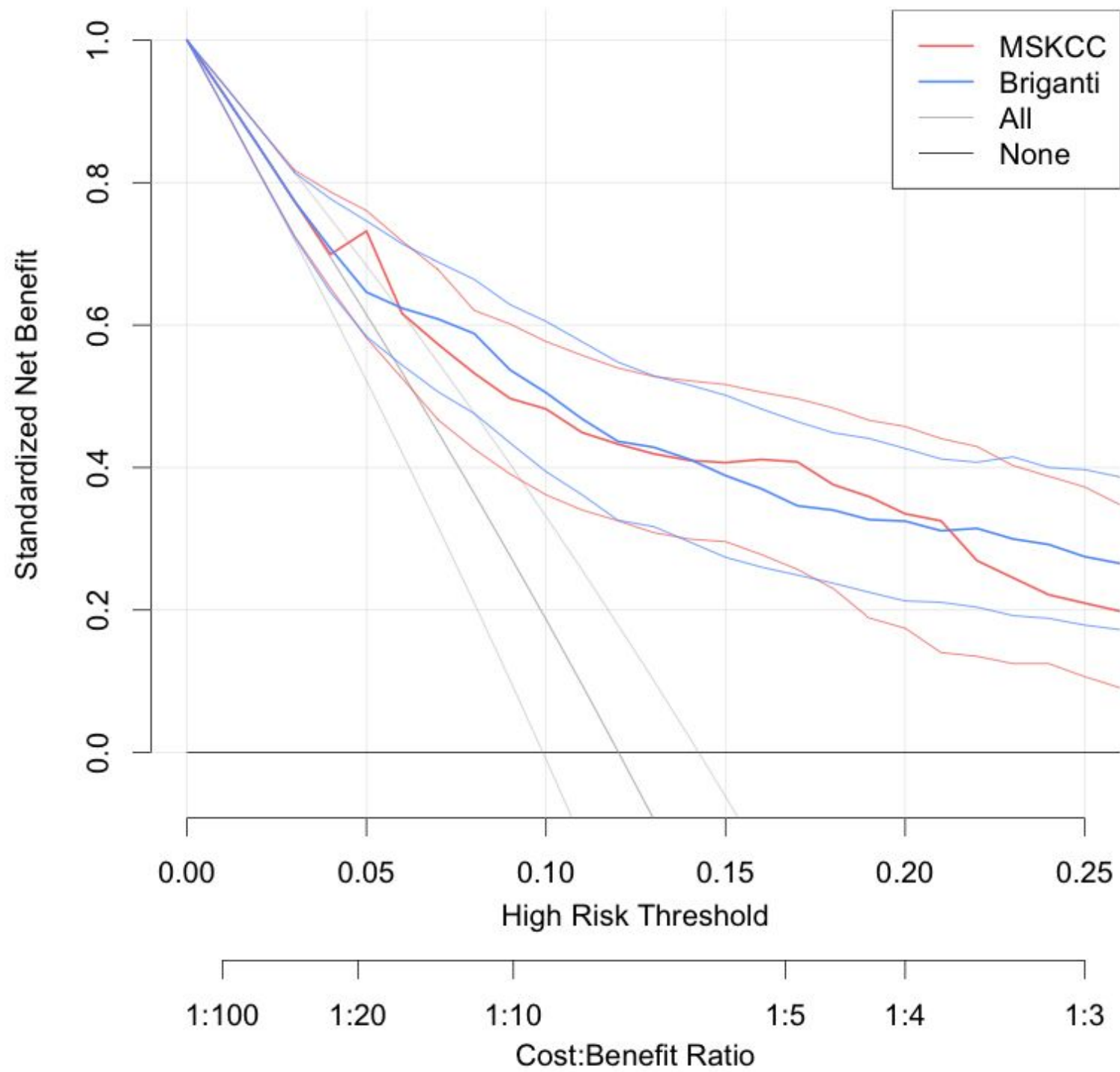

Supplement: Supplementary file 1 [file jcm-10-00999-s001.zip › Figure Nr. 3.pdf]

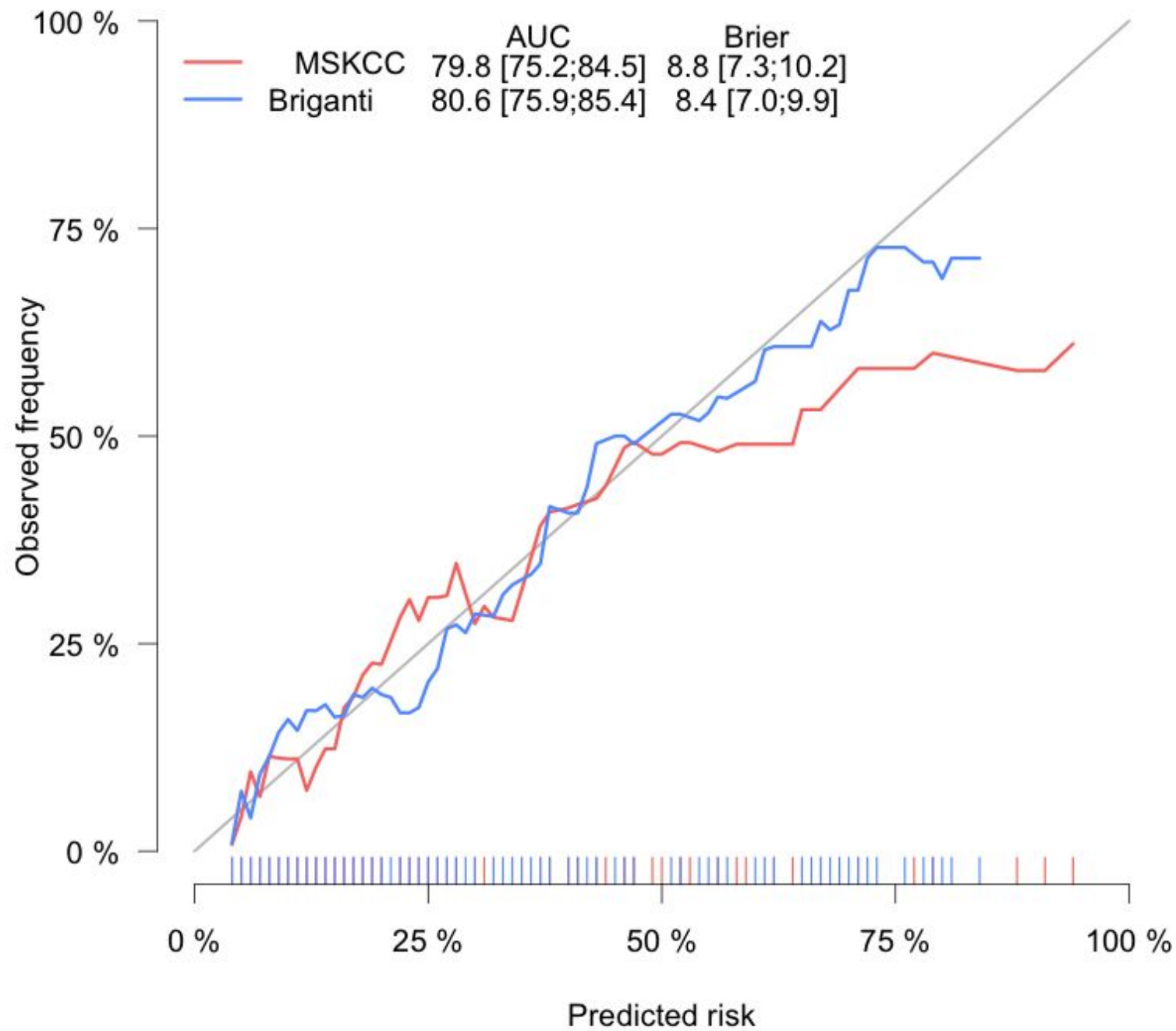

Supplement: Supplementary file 1 [file jcm-10-00999-s001.zip › Figure Nr. 2.pdf]

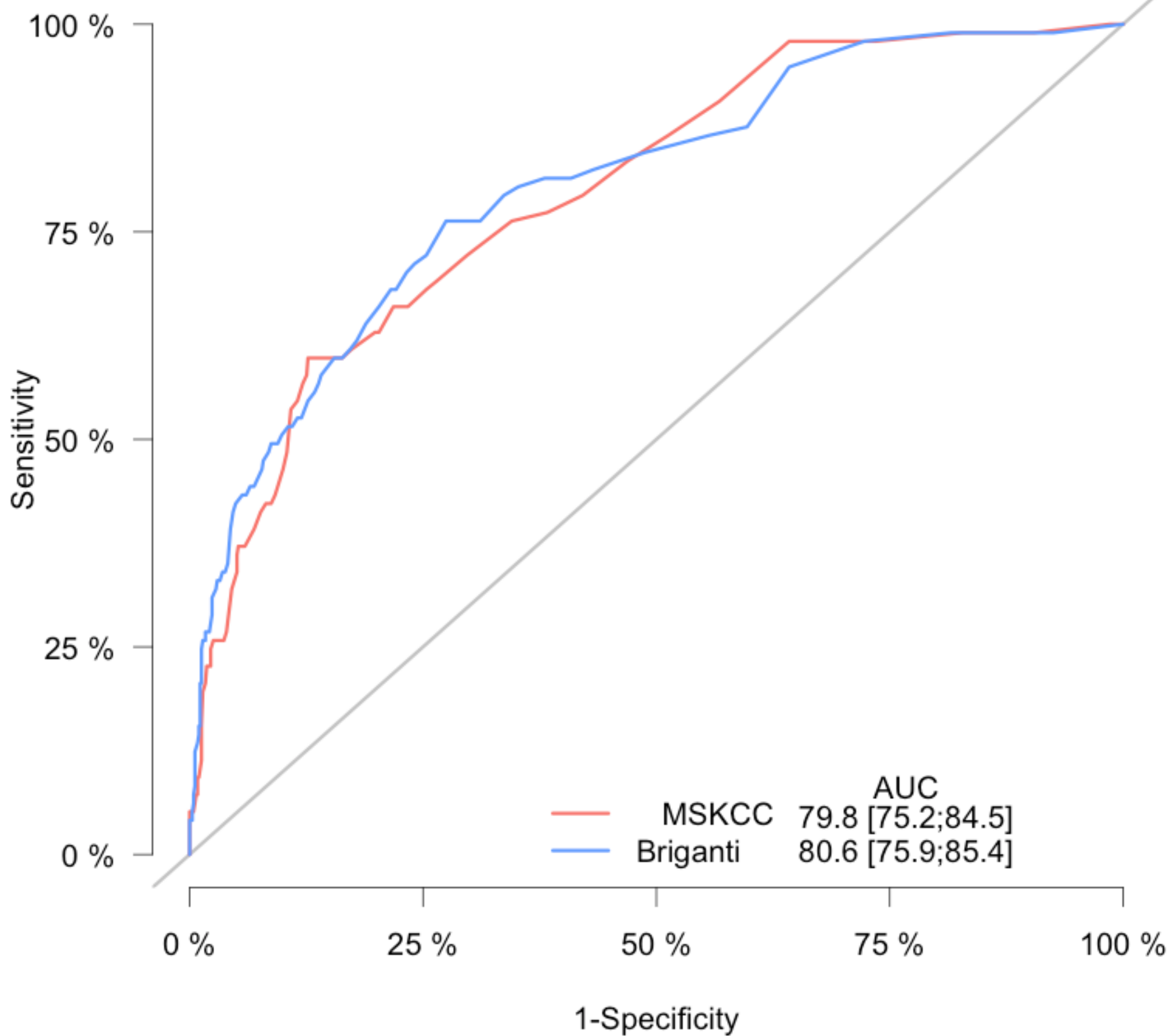

Supplement: Supplementary file 1 [file jcm-10-00999-s001.zip › Figure Nr. 1.pdf]

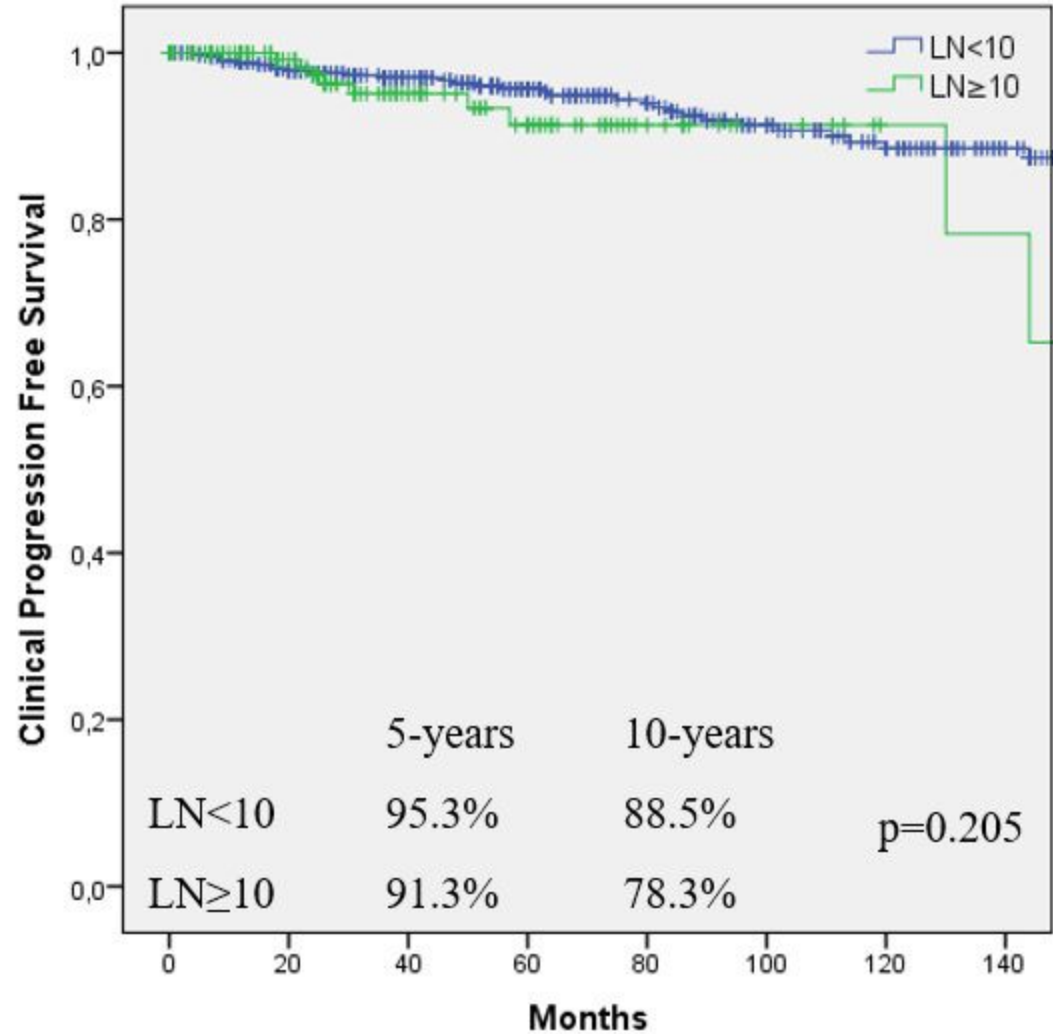

Supplement: Supplementary file 1 [file jcm-10-00999-s001.zip › supplementary figure 1 B.pdf]

Biochemical Progression Free Survival

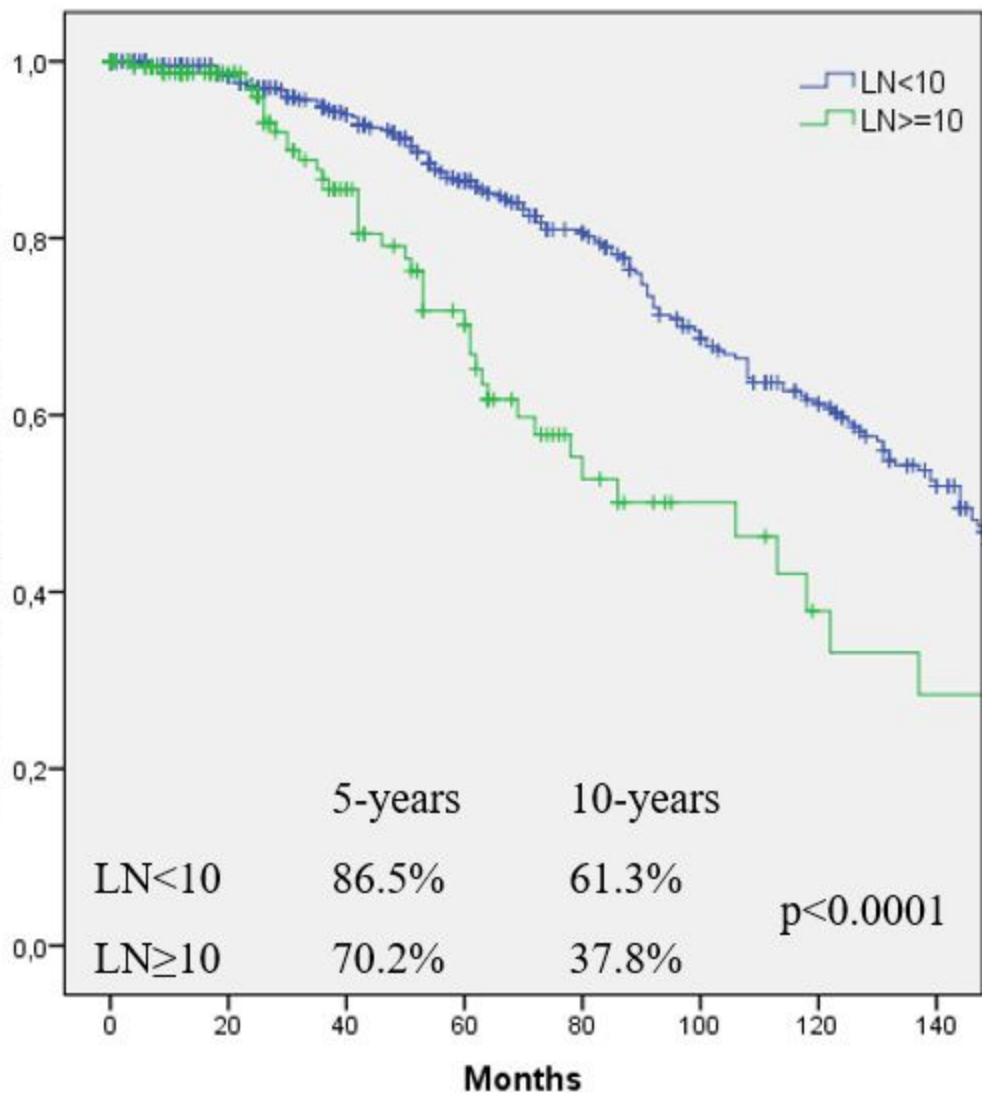

Supplement: Supplementary file 1 [file jcm-10-00999-s001.zip › supplementary figure 1 A.pdf]

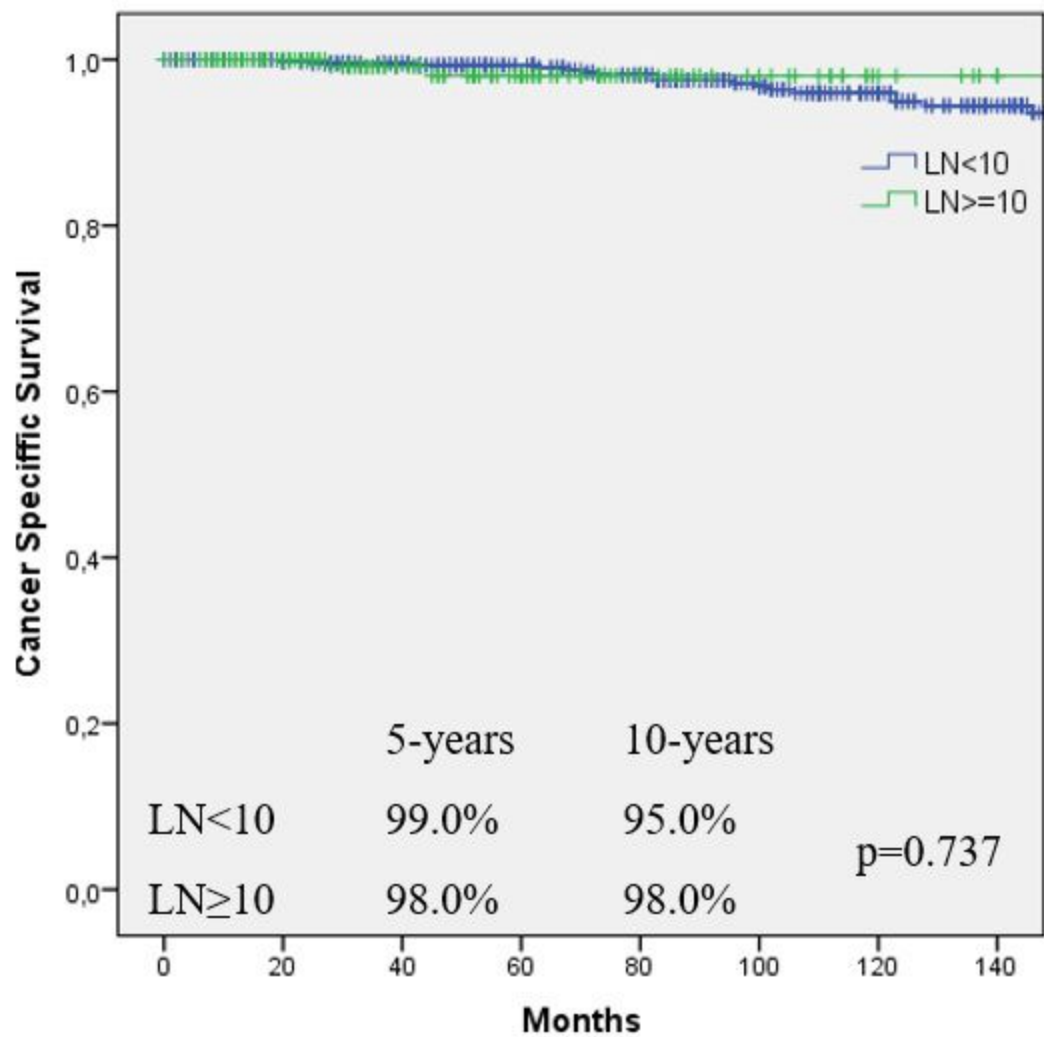

Supplement: Supplementary file 1 [file jcm-10-00999-s001.zip › supplementary figure 1 C.pdf]
